# Who should decide about children’s and adolescents’ participation in health research? The views of children and adults in rural Kenya

**DOI:** 10.1186/s12910-019-0375-9

**Published:** 2019-06-14

**Authors:** Vicki Marsh, Nancy Mwangome, Irene Jao, Katharine Wright, Sassy Molyneux, Alun Davies

**Affiliations:** 1 Kenya Medical Research Institute (KEMRI) Wellcome Trust Research Institute, PO Box 230, Kilifi, 80108 Kenya; 2 Centre for Tropical Medicine and Global Health, Nuffield Department of Medicine, Oxford University, Peter Medawar Building, South Parks Road, Oxford, OX13SY UK; 3 UK Nuffield Council on Bioethics, 28 Bedford Square, London, WC1B 3JS UK

**Keywords:** Africa, Kenya, Informed consent, Assent in children, Health Research, Children, Adolescents, Empirical ethics

## Abstract

**Background:**

International research guidance has shifted towards an increasingly proactive inclusion of children and adolescents in health research in recognition of the need for more evidence-based treatment. Strong calls have been made for the active involvement of children and adolescents in developing research proposals and policies, including in decision-making about research participation. Much evidence and debate on this topic has focused on high-income settings, while the greatest health burdens and research gaps occur in low-middle income countries, highlighting the need to take account of voices from more diverse contexts.

**Methods:**

Between January and March 2014, 56 community representatives and secondary school students were involved in eight group discussions to explore views on the acceptability of involving children and adolescents in research, and how these groups should be involved in decision-making about their own participation. Discussions were voice-recorded and transcriptions analyzed using Framework Analysis, combining deductive and inductive approaches.

**Results:**

Across these discussions, the idea of involving children and adolescents in decision-making about research participation was strongly supported given similar levels of responsibility carried in everyday life; existing capacity that should be recognized; the opportunity for learning involved; varying levels of parental control; and generational shifts towards greater understanding of science for adolescents than their parents. Joint decision-making processes were supported for older children and adolescents, with parental control influenced by perceptions of the risks involved in participation.

**Conclusions:**

Moves towards more active involvement of children and adolescents in planning studies and in making decisions about their participation are supported by these findings from Kenya. Important emerging considerations include the need to take account of the nature of proposed studies and prevailing attitudes and understanding of research in identifying children’s and adolescents’ roles. More research is needed to expand diversity and develop approaches to joint assent and consent processes that would fairly represent children’s and adolescents’ wishes and interests, towards their long term benefit.

**Electronic supplementary material:**

The online version of this article (10.1186/s12910-019-0375-9) contains supplementary material, which is available to authorized users.

## Background

Involving children and adolescents in health research is widely recognised as practically and ethically challenging, yet essential for the development of evidence-based health care in these age groups [1–3]. Traditionally, professional and societal attitudes have been conservative, taking children and adolescents as vulnerable by default; in need of protection by their parents and other responsible adults from avoidable life burdens or risks, and reasonably involved in research only under exceptional circumstances [4]. More recently, attitudes have shifted towards recognizing the critical importance of research involving children and adolescents, underpinned by evidence of high levels of unlicensed or ‘off-label’ medications currently in use in these age groups [1, 2]. Illustratively, the 2002 Council of International Organizations of Medicine Sciences (CIOMS) guidelines stated that ‘the involvement of children and adolescents in research is indispensable’ for research into diseases and conditions to which children are particularly susceptible [5], while the 2016 version of these guidelines states that ‘children and adolescents must be included in health-related research unless a good scientific reason justifies their exclusion’ [3].

Alongside greater recognition of the importance of child health research, there have been increasing calls for greater inclusion of children and adolescents’ voices in different aspects of the research process, from regulatory, scholarly and advocacy standpoints, as we go on to discuss in the next section. For example, Young Persons’ Advisory Groups (YPAGs) have gained popular support, particularly in high resource settings, as a means of incorporating age-specific input into research planning, including consent processes [6]. Recently, such a group was set up at a research programme in Cambodia, suggesting a wider acceptance of this role for children and adolescents [7, 8]. Within the debate on the role of children and adolescents in research processes, a main area of controversy concerns whether, when and how they should be involved in decision-making processes about their own inclusion in research [2, 3, 9]. While seeking informed consent from only parents or guardians is generally regarded as unacceptable, particularly for older children and adolescents, the exact processes by which they should be involved in decision-making remain a subject of debate.

As suggested earlier, a shift towards a greater role for children and adolescents in different aspects of research governance is primarily reflected in guidance and practice in high resource settings [10, 11]. At the same time, the highest burden of disease, disability and death in children and adolescents occurs in low-to-middle income countries (LMIC), where research resources are most stretched. In this paper, we argue that much greater attention is needed to questions around the active involvement of children and adolescents in research governance in LMIC contexts, noting that little empirical research has addressed this topic [12] or attempted to draw the voices of people from LMICs into the debate.

### Assent and consent for research involving children and adolescents

Internationally, individual voluntary informed consent is understood as a basic ethical requirement for the involvement of competent adults in health research. Competence is generally seen as a capacity (or set of capacities) that should be defined in relation to particular tasks [13] and – from a United Kingdom (UK) legal standpoint - as a situation of having ‘a sufficient understanding and intelligence to enable [a minor] to understand fully what is proposed’, and `sufficient discretion to enable him or her to make a wise choice in his or her own interests’ [14]. Since children become legally competent at different ages in differing jurisdictions, research ethics guidelines indicate that adolescents over the legal age of majority in a given context should provide informed consent for research participation, but that children under this age (both those capable of similar levels of understanding, and also those less mature but capable of giving a view) should be asked for their ‘knowing agreement’ or assent to participate [2, 3, 15, 16]. The concept of ‘assent for minors’ developed in response to concerns that parental or guardian consent alone would often be insufficient to support a child’s or adolescent’s ethical involvement in research, and as a way needed to ensure they were involved in decisions about participation.

Many challenges surround the use of age as an indicator of competence for decision-making about research participation for individual children and adolescents, even in relation to a given type of research project [17]. A core issue is that the skills, attitudes and behaviours underpinning ‘competence to decide’ are acquired at variable rates over time as part of a process of maturation influenced by environmental conditions, including the family and wider social context [9, 14, 17–21]. Differences in the age of maturity in different legal jurisdictions highlight this point. Competence may also be temporarily lost, for example, as a result of ill health and is not in any case an inevitable outcome of physical maturation in children. While recognising that particular levels of competence cannot be directly associated with specific ages, research ethics guidelines have generally responded pragmatically, through the use of age-specific categories, to the difficult task of regulating judgements on the extent to which an individual child or adolescent should be involved in decision-making about research participation.

Across high-income countries, there have been increasing calls to strengthen the voices of children and adolescents in research decision-making processes over recent years. Many such debates draw on legal guidelines for adolescent assent and consent to medical treatment in these settings, particularly referencing the concept of Gillick competence, for example as reflected in UK National Health Service guidelines [14, 22]. Highlighting increasing recognition that competence is a property that children acquire over time, influenced strongly by circumstances, Hein et al. [23, 24] in the Netherlands underlined the value of a case-by-case assessment of competence for research decision making in children over the age of 12 years, based on research using the modified MacArthur Competence Assessment Tool for Clinical Research (MacCAT-CR). Similarly, reflecting the need to recognize that decisions about research participation are not always equal in nature, the USA-based Society for Adolescent Medicine proposed in their 2003 revised Guidelines for Adolescent Health Research that adolescents should be assumed competent to decide about their own participation in minimal risk research, and that, for other forms of research, competence should be assessed on an individual basis and considered alongside parental permission [25]. The UK Royal College of Paediatrics and Child Health (RCPCH) published an ‘Infants’, Children’s and Young People’s Charter’ in 2017 to promote greater involvement of these groups in research, including individual decisions about participation as well as research agenda setting [11]. A recent UK Nuffield Council on Bioethics report (2015), a document that the research described in this paper fed into, supports the view that competence for decision-making emerges over time in children and adolescents, related to multiple factors including the type of decision and the wider social and cultural context [2]. Across many such analyses in high-income countries, support emerges for a ‘parent-child dyad’ as a unit of research participation decision-making, in the context of supportive family situations [2, 10, 11, 23].

There has been less focus on the issue of children’s and adolescents’ competence for research decision-making in LMICs. A notable exception is a paper from Cheah and colleagues (2012) from Thailand, who support the argument that age cut-offs are not useful or usable in their setting, and underline the need to include levels of risks involved in research in an assessment of competence for decision-making, and the risks of unfair exclusion [12]. At the same time, diversity of social context seems to have influenced national research ethics guidelines in several countries in Africa for some time, with the long standing inclusion of a category of ‘emancipated minors’ as persons under the age of legal majority (18 years) who are married, pregnant, have a child or are a household head [26, 27]. Such emancipated minors can make independent decisions about their own participation in research, but must demonstrate evidence of a good understanding of the research requirements [26, 28]. More recently, the debate on adolescents’ roles in research decision-making in many LMICs has been fuelled by recognition of rising levels of HIV/AIDS in these populations, and the need for research on HIV/AIDS prevention and treatment to include this age group and to take account of the diverse social contexts in which affected adolescents may find themselves [29–31].

In practice, one implication of taking a concept of emerging decision-making competence seriously is that there is a need to better understand the way that differences in children’s and adolescents’ lives, including in different parts of the world, might influence competence for decision-making about research participation. For international guidance, it is also important to understand how children, adolescents, parents and those in wider society might view proposals that this group should take greater responsibility for decisions about research participation in different socioeconomic and cultural contexts. The research described in this paper set out to explore the perspectives of adolescents and adults in Kenya on the involvement of children and adolescents in health research, particularly in decision-making about their own participation. In this way, we aim to contribute to local, national and international policy making and the wider literature on the topic of children and adolescents’ roles in research planning and policy.

The study was planned as a collaborative project between social scientists at the Kenya Medical Research Institute (KEMRI) Wellcome Trust Research Progamme (KWTRP), an international health research programme in Kenya, and the UK Nuffield Council on Bioethics [32], an independent body with international influence that examines and reports on ethical issues in biology and medicine. Between 2014 and 2016, a Nuffield Council on Bioethics working party, including two authors of this paper, addressed ethical issues for the involvement of children and adolescents in clinical research. Analysis in the final report was informed by the findings from the collaborative study reported on here [2].

### Community engagement and the KEMRI Wellcome Trust Research programme

Our study drew on an existing platform for community engagement within the KWTRP, a long standing large-scale international health research programme set up as a collaboration between KEMRI, Oxford University and the Wellcome Trust in 1989 [33]. The main base for KWTRP is in Kilifi County, a largely rural area on the coast of Kenya. Community engagement within KWTRP supports interactions around specific studies being planned, conducted or concluded as well as promoting understanding of health research and sciences more broadly [34]. Specific activities include offering information to and seeking advice from administrative, health and opinion leaders and various community members. Two particular engagement activities that this project drew upon were the KEMRI Community Representative (KCR) Network and the Schools Engagement Programme (SEP), specifically chosen to support discussions with groups of adults and a population of adolescents; these activities are described in the following paragraphs.

#### The KEMRI community representative network (KCR)

Many in-depth interactions between researchers and local residents in Kilifi involve members of a KEMRI Community Representative Network (KCRs). KCRs are local residents who are selected by their own communities to interact regularly with KWTRP community liaison staff and researchers to support planning and feedback around research. Currently, there are 220 KCRs working in 15 locational groups; every 3 years, new representatives are identified to take on this role. Community members are asked to choose representatives who are typical of their location; the only restriction is ensuring gender balance. Recent surveys indicate that KCRs are reasonably typical of the wider population, although a higher proportion have secondary schooling experience than average.

#### The Schools Engagement Programme

A broad approach to fostering understanding and interest in science and health research in Kilifi is taken through the Schools Engagement Programme [35, 36]. SEP was developed through a participatory action research approach involving the County Education Office, secondary school principals, teachers, students and their families and research staff. At the time of this research, SEP had involved 20 secondary schools in Kilifi, including through in-depth activities (such as lab tours, careers talks, support for national school science competitions and internships) and broader-based programmes (such as a web-based interactive information-based game “I’m a scientist, get me out of here!” Kenya) [36]. While primary education in Kenya has been state-sponsored since 2003, at the time of the study secondary education was fee-based and academically competitive. As a result, around half of students leaving primary school took up places at secondary schools [37], some with academic scholarships; their distribution across available schools depending on academic performance at primary school leaving exams. Given the distances that students may have to travel, many secondary schools have partial or full boarding requirements, while others function as day schools.

## Methods

The study was designed as a qualitative exploration of the experiences, views and reasoning of a group of 56 secondary school students and adults who were resident within the area surrounding KWTRP in Kilifi County. As described in the following sections, the methods used were based upon discussion and structured debate within a small group format, drawing on a deliberative approach towards exploration of views around the involvement of children and adolescents in different types of research in this social and geographic context [38, 39].

### Site and participant selection

We talked to a total of 33 KCRs and 23 students in eight group discussions, conducted between January and March 2014 (see Table 1 for a detailed list of participants). KCR discussions were held as part of regular meetings with community liaison staff, taking the form of natural group discussions with up to 10 participants. The four KCR groups included in the study were purposively selected to reflect diversity in urban/rural status and to include KCR groups with smaller total membership (10 or less) to ensure sufficient individual participation. As Table 1 shows, groups included roughly similar numbers of men and women (a criterion for KCR group selection), as well as a mix of levels of exposure to formal education, ages and religions.

**Table 1:** List of participants and characteristics

Group type	Number (Female)	Age (years)	Years schooling	Rural/urban home	Religion^a^		
					C	M	T
Girls’ boarding school	6 (F=6)	16 -18y (median 16.5y)	11y	1/5	3	3	0
Mixed day school	6 (F=0)	18-20y (median 19y)	10-11y	1/5	3	3	0
Girls’ day school	6 (F=6)	16-17y (median 16y)	9-10y	0/6	4	2	0
Boy’s day school	5 (F=0)	16-19y (median 17.5y)	9-10y	0/5	2	4	0
KCR 1	8 (F=3)	36 – 60y (median 44.5y)	4-12y (median 8y)	8/0	8	0	0
KCR 2	9 (F=4)	32 – 59y (median 41.5y)	8-16y (median 12y)	9/0	8	1	0
KCR 3	8 (F=3)	30 - 55y (median^1^ 38y)	0-12y (median 7y)	0/8	6	0	1
KCR 4	8 F=(5)	26 - 80y (median 51.5y)	7-16y (median 10.5y)	9/0	4	3	1

^a^*C* Christian, *M* Muslim, *T* Traditional

Within SEP, four schools were purposively identified to reflect diversity in makeup, including a girls’ boarding school, a girls’ day school, a boys’ boarding and day school; and a mixed day school. Within schools, participants were chosen from the middle two year groups, Forms 2 and 3. Participants were aged between 16 and 18 years, as shown in Table 1. Individual students were selected by form teachers, who were asked to bring together a diverse group including a mix of religions and academic interests, but positively selecting students likely to contribute to a group discussion of this nature. Based on experience in SEP, each focus group included only boys or girls to promote open discussion.

### Data collection and analysis

KCR groups met at their normal meeting places, often the offices of local administrative leaders; students’ discussions were held in a classroom in their schools. Facilitators led discussions using the topic guides shown in Additional files 1 and 2, which included the introduction of and discussion around three different types of research: a study based on a non-sensitive questionnaire only; an observational study involving the collection of blood samples; and a clinical trial. Discussions lasted about one hour, and were held in Swahili, a Mijikenda language, or a mixture of Swahili and English. Facilitators were social scientists at KWTRP with experience of moderating group discussions. For SEP groups, a note taker accompanied the facilitator, and at KCR groups one or two community facilitators were also present, allowing for discussion and documentation of key points during and after each meeting. NM and IJ collected data from students’ discussions, and IJ and VM from KCR meetings. After each discussion, the team held debriefing discussions and made summaries of key points to feed into subsequent discussions. Group discussions were voice recorded, transcribed and translated into English.

We used a Framework Analysis approach [40] that included steps of i) immersion in the data (NM, IJ, VM and SM); ii) development of an agreed initial coding framework through discussions between NM, IJ, VM, AD and SM on data from three diverse transcripts, supported by considering data from discussion summaries; iii) a coding (or indexing) process, led by IJ and NM; iv) the development of analysis charts to capture data around key areas included in the discussion guides or emerging issues, by group; v) interpretation of data in analysis charts by synthesizing findings across groups and relating these to the research ethics literature, including that on assent and consent processes in minors (all authors). While the whole dataset covered a broader range of topics (particularly the acceptability of involving children in research), as shown by the topic guides, in this paper we focus on describing our findings on perceptions of the role of children and adolescents in making decisions about their own participation in health research.

### Ethical considerations for the study

For a study that aimed to explore adults and adolescents’ views on issues around their involvement in research in general, we were very aware of the need to carefully consider our own consent processes for this study. In doing so, in addition to awareness of the literature we have already described, we considered the experience gained during earlier social science studies at KWTRP involving secondary school students, conducted as part of evaluating SEP itself. Given the age of the adolescents involved and the minimal risk nature of our research [3, p12], including the use of focus group discussions planned in collaboration with head teachers to ensure minimal disruption to students’ learning, we moved forwards with a process in which students were asked for verbal consent to participate in discussions. After their initial agreement, we ensured that individual students understood that they were free to choose when to join in discussions, and re-visited schools to give feedback on our findings to head teachers. Verbal consent was also sought from KCRs for their participation in the study. The research was approved by the Kenya Medical Research Institute Science and Ethics Review Unit (SCC 1463), and supported by the County Director of Education, school principals and class teachers.

## Results

Across these group discussions, there was a general sense of interest in the topics, for both KCRs and students. Students in particular often had strong views to share, and talked animatedly amongst themselves and to us. In this paper, we present findings on perceptions of the role of children and adolescents in making decisions about their own participation in health research. Across all groups, there was wide and strong agreement that in principle children and adolescents should be involved in decision-making about their own research participation, in degrees related, firstly, to the implications of their participation and, secondly, to their ability to make a reasonable decision, as will be discussed in the following sections. In general, people’s reasoning around *why* involving children and adolescents in decision-making was important emerged as part of explaining *when* and *how* they should be included, particularly in discussions about maturity and age.

### An influence from the perceived implications of research participation

For KCRs and students, views on how much control children and adolescents should have in research decision-making were linked to perceptions about their levels of maturity and ability to make ‘good decisions’. While characteristics and relative ages of maturity are discussed in the next section, most fundamentally, a ‘good decision’ was seen as one that supported the welfare of the child or young person, or at least did not undermine this. Where participants in this study saw that health research was itself intended as a public good (this was the case for most KCRs and for many of the students) decisions to participate to ‘help others’ were also seen as in principle ‘good’, but not at the cost of the individual child or young person’s welfare. Altruistic ideas were particularly put forwards by students, some of whom expressed excitement at the idea of participating in research:

Okay [if] it comes to a good conclusion, like you get the vaccines, I will be like “Yeah, my blood sample was taken in the research!” so I will be excited (student, female, 16y)

As a consequence, KCRs’ and to some extent students’ views on the extent to which children and adolescents should be listened to *when their views differed from those of their parents* were directly related to their perceptions of the social value, risks and benefits involved in the child’s research participation. While KCRs often saw older children and adolescents as able to act independently in many ways, their assessment of the risks of participation in any particular study as being ‘too high’ would trump even an older teenager’s wish to participate. In this way, children and adolescents were seen as much less likely to be able to make good decisions *on their own* about participation for research perceived as more risky (such as clinical trials) or burdensome (such as studies involving blood sampling); and much more likely to be accepted as independent decision-makers in relation to low risk studies (such as those based on questionnaires).

I can say involving the child [in decision making] is okay, but it depends on the type of research and also the age of the child. For example, my younger brother [aged 13 years] was involved in a study and was given a diary to fill in for three days… they asked me if I can agree, and I told them he himself can decide because there’s nothing there, it’s just talking and filling. He said “I will do it”. You see? He is a child, but can express himself (KCR, female, 26y)

If maybe you are taking part in research on maybe a vaccine, that is when you have to consult your parents but if it’s about blood sample, urine samples, answering questions, you can just decide for yourself (Student, female, 17y)

Showing the perceived importance of the welfare argument, where KCRs felt that an unwell child would benefit directly from participation in a study thought to include important medical benefits, the parent’s view would again be seen to reasonably overrule a refusal on the child or young person’s part. Many students agreed with this position, with caveats about age and the perceived benefits of participation. Some students added an additional concern about taking decisions independently of their parents in case adverse consequences followed, when parental support would be needed. In this way, parental approval was seen as a form of ‘insurance’ against future problems.

Variation in these opinions reflected differing assessments of risks and benefits, as well as of the child or young person’s capacity to make the assessment. Underlining the importance of communication in research, some perceptions of risk and benefit were highly exaggerated, including unrealistic hopes for how quickly research might lead to effective new drugs or vaccines being locally available and fears (particularly amongst students) of drastic levels of harm, often associated with lack of understanding of research governance processes:

Maybe let’s say from a few months up to around four to five years…if a vaccine is harmful, you will end up losing millions of adolescents coming up in the next generation! (Student, male, 19y)

Like me now my child, let’s say, you have to confirm to me things like that, “your child will not die”! As in you should also tell me in case it doesn’t work, what effects will be experienced. As in you just have to let me know. (Student, female, 17y)

### The ability to make a reasonable decision: age and competence for decision-making

In conversations about competence for a given type of decision (for example, participation in a particular study), the qualities most commonly described were the ability to communicate and reason (for example, illustrated by expressing views and asking good questions), understanding of self (such as not being easily swayed by others), undertaking other similarly independent activities (often, walking to school alone) and understanding of what was being proposed, including the implications of participation.

Or for example, she has seen her friends doing it…eeh, she says “I will do it!” She doesn’t know herself, she hasn’t understood herself. (KCR, female, 49y)

The 9, 10 year olds…somehow they do understand themselves, if the parents have agreed they should go for that research, they also should be asked if they want to. (Student, female, 16y)

I don’t think it’s 18 and above or less than 18 … you have to use your intelligence, if you consider the advantages and think it is going to help, it’s you who will make the decision, you don’t have to quote the age or something. (Student, male, 17y)

While age in years was generally mentioned in talking about competence, participants clearly perceived wide variation in the ages at which various capacities develop in children. However, there was general agreement that as children matured, they should become more involved and have increasing levels of control in research decision making:

[Talking about competence in older adolescents] …we have this one who has just been born…zero to five… who is yet to start speaking. But there’s the one who walks alone to the nursery school, for this one, she can make a decision, but her capacity for decision making is still very low…But now I was reaching out to those who have started maturing a bit…fifteen or seventeen years, I was talking about these ones now (KCR, male, 37y)

Views expressed by KCRs and students were largely similar, although students’ assertions of their independence in making these decisions were more commonly and strongly made.

#### For very young children

For infants and children up to the age of about two or three years, there was wide agreement that children could not express themselves or understand what was happening around them well, but were dependent on their parents for all their needs. The younger the child, the more dependent they would be. At this stage, it was not seen as important or even possible to involve the infant or child in decision-making, which should only involve their parents:

For example the child has been born today, there you the parent are given an explanation and you decide everything…you as the parent knows the importance of that research (KCR, male, 32y)

#### For older children

KCRs and students talked about an older group of children, with ages identified variously between four and 10 years, who were seen as having sufficient capacity to be involved in the process of decision-making, although not in making a final decision. Where their parents had already taken a decision that the child should participate, the type of involvement was described as explaining research to the child in a reassuring way that would convince them to participate.

When she is five, six years onwards…you have to soothe the child a bit so that she be involved in that research (KCR, male, 32y)

For older children, these explanations often included an idea that the child should be brought to understand why participation was important, serving as both persuasion and ‘education’, where the latter was related to research and to promoting their own autonomy:

The 9, 10 years olds there… somehow they do understand themselves, so if the parents have agreed they should go for that research, they also should be…told the benefits of having such kind of a research, they should be educated somehow. Somehow they will end up understanding and making a decision also. (Student, female, 16y)

Many KCRs saw challenges in trying to convince children around this age to participate, particularly for studies involving blood or other forms of sampling. Some described uncertainty and were very unsure about the best way to do this, with age again an influence. It was often seen as reasonable to restrain a younger child while, for example, blood samples were taken – particularly if this was a minor and/or familiar procedure (such as taking a finger prick blood sample), and the research seen as important, including that the child would directly benefit. For older children, or less individually beneficial procedures, the majority saw it as neither desirable nor feasible to force children to participate, implicitly describing a right to refuse:

To about three, four [years] there, I will decide for her. But when the child is a bit older, and she says “No, I don’t want…it might hurt” and I see that it’s not a must because she is not even sick [I will say] “okay, then go and play with your friends”… she might even run away. Should I run after her because of research? (KCR, female, 26y)

#### For young adolescents

With increasing maturity and around early adolescence, most KCRs and students felt that children and adolescents should be centrally involved in making decisions about participating in research. This age group was again defined differently, but largely fell within the range of 10 to 14 years of age, with some including children down to six years.

The key characteristic underpinning perceptions of increasing competence for research decision-making at this stage was that children and adolescents had already begun to lead relatively independent lives. Many would make their own way to school, often walking long distances, and had relatively little day time contact with parents. They were often given greater responsibility for more complex domestic tasks, including independently taking care of younger siblings. It was assumed that these routine daily experiences would give children more capacity to make other decisions of comparable complexity.

If he can answer question, let him just answer. Sometimes they do things which the parents themselves get surprised, because they know how to do things…if at thirteen years he goes to Mombasa, Malindi, and back, by himself, if you ask him silly questions, won’t he surprise you with his answers? (KCR, male, 55y)

Some suggested that these forms of increased responsibility would also make it difficult for children to accept being completely under their parents’ control. In addition, KCRs and students talked about a generational shift in life experiences between children and their parents at the same age. Part of this shift was linked to formal learning, since children more commonly attend school than in the past, a process accelerated by the introduction of government sponsored primary education in 2003 [41]. Many older children and adolescents were also seen as having a wider - often global - field of influence and learning, through access to the internet, for example using mobile phones and internet cafes.

Children nowadays, they call themselves ‘digital’, they are too difficult! You can decide for that small child, let’s say from 0 to 10 years, but from 11 onwards, heh, there’s difficulty! You have to sit with the child and talk to them, when they agree, that thing can be done, if they refuse, so be it. (KCR, male, 46y)

At the same time, this stage of maturity was still characterized by limitations to children’s capacity to understand all that was being proposed, and a tendency to change opinions easily as a result of peer influence or other forms of persuasion. The type of research involved (or more directly, risks and benefits of participation) became a critical deciding factor for the extent to which the child or young person should control decisions, as described earlier.

Even more than for younger children, it was not seen as either reasonable or possible to force children of this age to participate against their will, unless there was some reason to believe the child would gain important health benefits from participation, that is, for seriously ill children. One male KCR noted that the revised Children’s Act in Kenya [42] made it clear that children could not be forced to participate in research against their will, as this would be in breach of their rights to be protected from emotional or physical abuse, with potential legal consequences for parents. Overall, at this stage it was seen as particularly important that parents try to explain the research to their children, including trying to convince them if they thought the child should participate, again often illustrating a pedagogical component:

Those ones from 10 years onwards should come and we sit together, but any decision should be from them…we will explain its advantages. “Because even these drugs you use, there are children like you who were involved [in research earlier], so you also accept. These researchers work with the hospital so if there are any side effects, you will get treatment”. Those from 11 onward, those should make their own decision, ours as parents is to try to help them (KCR, male, 45y)

#### For older adolescents

In general, KCRs and students felt that adolescents at around 16 years and above were capable of making their own decisions about research participation, but many recognized that parents still had a role to ‘sit and help’ adolescents understand what was being proposed, and some might prefer their parents’ support in making this decision. Two main ideas underpinned these views: firstly, that at this age a good decision might more often need to be weighed up by the young person and their parents together; and, secondly, that the young person should be allowed greater control in decision-making by virtue of their increased maturity and independence. The underlying assumption here was of a progressive move towards competence in decision making, such that older adolescents could be trusted to take responsibility for their own welfare. ‘Generational shifts’ described earlier were particularly important influences here, with older children and adolescents seen as potentially having a better understanding of science and research than their parents.

So their child [young person] has been explained [to] and understood, but the parents, because they haven’t really understood, their child will have to explain to them. “You know what it is, dad, the benefits of this research is this and this and this.” In fact he can be a good teacher to educate these parents of his until they agree. (KCR, female, 37y)

Students’ discussions of decision-making were particularly informative since most were themselves in this age group (see Table 1). Most students felt they would not be strongly influenced by parents’ views, but saw their own assessment as most important. The young person would then try to persuade their parents to agree before taking a decision to participate.

Let’s take an example of me, I’m 17 years, I understand, I can give my personal opinion without involving my parents. I’ll accept if I see it will be important to me. If my parents know too but they are denying me, I’ll try to educate them because maybe it will be due to lack of knowledge. I’ll have to educate my parents so that when we leave there, we’ll be in agreement. I cannot leave without their permission, they are my parents, I must inform them and agree with them. (Student, female, 17y)

Parental influence was often discussed in relation to the parent’s knowledge and experiences:

It depends, if your mother is a lawyer or something obviously if you refuse, she won’t tell you to participate. But if you take a parent with no education, who has lived a life of ‘old times’, when she’s just told something she can say ‘let him go’. So you will have use your brain there … if you have refused, you have refused. (Student, male, 17y)

As described earlier, this view of greater independence was tempered in some cases for research seen as having significant risks, where many students felt that their own positive views on participation should be checked and approved by their parents. But, at an extreme, a few students - both boys and girls – described their parents’ decision as being more important than their own. This acceptance of parental authority was based on respect, recognition of parents’ greater life experience of life, and trust that parents would only act in the interests of their children:

I’ll agree because the way I know myself since they started bringing me up, my parents can never want any bad thing to happen to me. My parents love me! I’ll go [participate] because a parent can never allow bad things to happen to their child. I’ll respect my parents and go. (Student, female, 17y)

Parents have seen the sun earlier…I mean she knows a lot…she has experienced a lot… so whatever she tells you, you can also think well about it, that parent loves you unconditionally, she can never have bad intentions for you. (Student, male, 18y)

At the opposite extreme, a few students rejected the possibility of parental influence at all. In this case, students would either inform their parents after agreeing to participate or conceal their participation from parents.

For example, if my parents are not that educated, they won’t understand what the research is about… but I’ll know the importance of the research. For me, I’ll participate. If they don’t understand, I’ll have to hide it from them. I won’t tell them! (Student, female, 17y)

Additional reasons students used to justify taking decisions independently were based on comparison with other areas of life in which they take decisions alone, listening to but not necessarily taking account of their parents’ advice. These included situations involving risks, including through sporting activities.

### The value of talking together and challenges for parental support to decision making

As these findings show, many KCRs and parents ended up talking about *the value of parents and adolescents talking together* to make a good decision about research participation. The young person was seen to have the central role in decision making but parents might be able to support them to think through the advantages and disadvantages of participation.

If you think your parents are going to have a different decision from yours, you ought to sit down and if your parent is understanding he will involve you in decision making, he cannot leave you and make the decision alone. You will sit and share your views and at last come up with a solution that is going to be binding (Student, male, 18y)

Therefore, throughout discussions, students and many KCRs regularly stressed the need for researchers to provide good information to adolescents to enable them make ‘good’ decisions on their own.

In ascribing a role to parents in making or contributing to decisions about research participation for their children, KCRs and students described situations where parental opinions could not easily be fed into this process. Some situations might be more particular to this context, while others were more clearly generalizable. These included situations where parents do not agree with each other; and where adolescents attend boarding schools at some distance from home.

#### Where parents don’t agree

When parents were thought to have primary responsibility for making decisions about their child’s research participation, it was generally felt that the parents should discuss and come to an agreement together. Mothers were seen as having particularly important roles in making decisions about research participation in younger children, based on the traditional roles they have in bringing up children, including responsibility for their health. But, in this traditionally patrilineal community, fathers often have primary control over family resources, giving them the role of primary decision makers for issues affecting use of these resources, including research participation [43].

Given this situation, there were many practical challenges seen for joint decision-making processes for parents. Children are often recruited into studies when they attend health facilities with their mothers during the course of an illness, in which situation fathers may not be present, and mothers may have travelled long distances to reach the facility. In addition, in this community, fathers often work away from home for periods of time, and would not be easily reached. In any case, any agreement reached between parents would be likely to primarily reflect the father’s views. Although traditional family dynamics have shifted to reflect greater economic empowerment of women within this and other areas of Kenya, the expectation that a father should consent to their child’s participation in research is still common. This was often described as a form of insurance; that the repercussions for the mother of deciding on her own that a child should participate in research could be very serious if anything subsequently went wrong. In this situation, a mother could suffer serious hardship, including being ‘sent back’ to her parent’s home [44].

Parents and students felt that the views of older children and adolescents could in fact be drawn upon to settle any disagreements between the parents, if such a process could be set up; a view that notably was supported by some students who had in fact reached an age of majority:

If the parents are different, maybe dad may want, mum may not.… I think the best thing is to sit with your parents and explain to them, then the parent who is on your side can also explain to the one refusing… maybe from there you will have made your decision and told them everything, isn’t it? (Student, male, 20y)

In addition, students saw researchers as having a key role to help parents to make a decision when there was disagreement, being more expert than either the parents or the young person in relation to the research proposed.

#### The case of boarding schools in Kenya

In the KWTRP, several studies have centred on primary and secondary school students, run in collaboration with the County Education office. There has been lack of clarity over appropriate consent and assent processes in this situation, particularly the role of school principals and other senior teachers. For day school pupils, KCRs and students felt that the role of parents and their children in decision-making about research participation would be no different to the general situation; educational professionals should not act *in loco parentis*, and the child’s assent would be essential in addition to parental consent. In the boarding school situation, which as described earlier is quite common amongst Kenyan secondary schools, communication with parents is more difficult. In this situation, KCRs and students were clear that teachers and principals at secondary boarding schools should have an important governance, information-giving and advisory role but not be able to give consent for a young person’s participation in school-based research. Instead, either the young person should decide on their own, for example for low risk research, or the young person or researcher (with the young person’s permission) should communicate with parents to obtain their consent for the student’s participation.

### Gender of the young person as a potential influence

In some student groups, gender was described as an influence on the ways in which decisions about research participation within families were made. For a few female students and KCRs, the risks of participation were seen as higher for adolescent girls than for boys of the same age, linked to concerns that fertility or unrevealed pregnancies might be affected and that blood sampling was less safe in girls who were already experiencing regular menstruation. Others, particularly but not exclusively from Muslim families, described that parents were likely to protect their older daughters more than their sons from outside influences in general, leading to unwillingness to allow girls to be involved in research, particularly if this meant travelling outside the home and/or being in the presence of men outside the family. A young woman who wished to participate in research might therefore be less likely to be listened to by her parents. In fact, this protective attitude was described as increasing with the age of the child, affecting the movements and decisions of older more than younger girls. As a Muslim student described ‘the older you grow the more difficult it becomes to get out of the house’ (Student, female, 16y). Her fellow female student, a Christian, added:

I think those who give permission [parents] can mostly allow a very young girl child, but if it’s a big girl of like 16 years, they’ll [parents] find it difficult to let her go, when people come and say they want to go with her to do some research, because obviously they’ll think in a negative way….they are not up to any research procedures whatsoever but they have their own missions! (Student, female, 17y)

### Trust and communication, including on clinical research and care

As has been described, participants commonly reflected on the importance of parents and children fully understanding the nature of any proposed research, particularly the goals, procedures, risks and benefits to support reasonable decision-making dynamics between children or adolescents and their parents. Researchers were seen to have a core responsibility to give clear and comprehensive information, including about any ‘side effects’ to look out for, both those that could be minor and temporary, and those which might be more serious and that the parent should act upon. While these areas are clearly the focus of individual informed consent processes, in communities where there is little exposure to research more broadly supportive and interactive engagement processes involving the wider community are likely to be needed [45].

A particular issue for decision-making dynamics and communication emerged over difficulties in distinguishing between clinical research and routine health care, particularly where (as is common in the KWTRP) these were being run by the same personnel and in the same place [45]. Where clinical research was interpreted as routine health care, parents would generally support whatever researcher-physicians proposed, and be unlikely to allow their child to refuse or to influence their decision. This risk of conflating clinical research and care, sometimes described as a therapeutic misconception, has been a driver for components of the community engagement programme at KWTRP, aiming to build understanding of the differences between these and promote informed choice in research participation [46]. In practice, the difference between clinical research and care – and the way this should affect decisions to participate – can be difficult to separate. In many instances, care offered during research was not so much confused with clinical care as perceived as of higher quality and more reliably available than that through over-stretched public health resources:

I will accept [for child to participate in research]…when we go back to a child who is sick and I take him to the hospital for example, and the way we understand that research is voluntary, so you will be asked those questions. And when he is being researched on also he will get some treatment and at the same time research will be going on. The outcome of the research might teach us which medicine has cured the disease and won’t that help us in the future? (KCR, male, 57y)

Effective communication about these complex areas is likely to be key to building parents’ and older children’s confidence in making a decision about whether or not their child/they should participate in studies. The development of trusting relationships between health workers / researchers and potential participants / parents would also be key to building parents’ and older children’s confidence in making a decision about whether or not their child/they should participate, as suggested by quotes from two KCRs in this study:

Like [XX - name of a community facilitator] and [local area]… now maybe my child has been given those drugs and she took it, knowing XX will come, “How is she doing, no problem?” “No problem. She is doing well” and he passes by. Then we know we have someone in our midst who cares for us. (KCR, female, 52y)

When they came the first time, they explained to me about research on pneumonia. By then I had not understood KEMRI and its roles, so my heart was a bit hard there, and I said that my husband was not around so perhaps he should come and I tell him first (KCR, female, 36y)

We have argued elsewhere that, while the nature of relationships built between community members and researchers is a critical influence on the effectiveness of communication about research, it will also be important to ensure that the forms of trust built within relationships are well-founded, so that choices are not made on ‘blind’ forms of trust in individuals or institutions [18]. These quotes show a risk that trust may be unduly inflated by familiarity, and that communication and relationship-building over time can change levels of trust. In these ways, the design of engagement processes is critical to supporting informed and free choices about research participation, and may be particularly important in more complex joint consent processes.

## Discussion

Against a background of a shift in attitudes in international research ethics guidance towards greater involvement of children and adolescents in decision-making about research participation, this study has provided relatively novel evidence of the attitudes, perceptions and recommendations of a group of secondary school students and adults resident in a rural Kenya setting around this area of debate. One value of these findings is in contributing to understanding grounded perspectives from a LMIC context; voices that are currently lacking in debates about children’s role in research decision-making.

Overall, we show that the attitudes of students and KCRs in Kilifi were supportive of such a shift, while directly raising caveats in relation to the type of research and the actual maturity of children or adolescents. The primary arguments drew on recognition that: i) older children and adolescents carry levels of responsibility in their everyday lives that are similar to the decisions they would make about participation in some research, as had been argued from a South East Asian context [12]; ii) that children and adolescents already have the capacity to do this, which should be respected; iii) that even quite young children as well as older ones could benefit through learning from their involvement in decision-making; iv) that parents may not in practice be able to control their older children’s decision; and v) that older children might in some cases have more knowledge about the areas of decision-making than their parents. The main arguments also lend support to the idea that, at least for older children and adolescents, consent processes should generally involve both the parent(s) and their child, working together, rather than either of these on their own. A common phrase used to reflect this idea was the need to ‘sit down and talk’ together about such decisions.

In this way, across our findings, we note KCRs’ positivity towards, and students’ insistence on, active roles for older children and adolescents in research decision-making. These views may reflect changing attitudes across recent decades in many sectors of Kenyan society towards the roles that children and adolescents are expected to play in the family, in terms of obedience towards parents and adults [47]. A key legal document underlining increased recognition of children’s rights in Kenya is the 2017 revision of the Children’s Act 2001, which defines children as ‘human beings under 18 years’ [42] (p11). Mirroring similar language in the United Nations Convention on the Rights of the Child (UNCRC), the Kenya Children’s Act makes clear that ‘a child shall be entitled to protection from physical and psychological abuse, neglect and any other form of exploitation’ [42](p16) [48]. As shown in the findings section, this protection was recognized as an influence on the extent to which parents would be able to ‘force’ children or adolescents to take part in research, against their will, including the possibility that an adolescent might make a legal complaint against parents in such instances.

For many of the arguments made, there seems to be a direct implication for policy, but in some cases there are additional ethical considerations. We discuss these issues in the following paragraphs, taking forwards our findings on why children and adolescents should be involved in research decision-making; whether their views should ever be overruled by their parents; and when and how they should be involved in these decisions. In the last section, we discuss a key emerging cross-cutting issue of the risk that parents and their older children might conflate health research and clinical care, undermining the validity of decisions taken about participation, particularly for research involving unwell children and adolescents. We therefore underline the need to strengthen understanding of research across relatively research-naïve communities as part of novel approaches to consent for children and adolescents. Before this, we consider limitations to this study and our interpretation of the findings.

### Limitations to this study and our interpretation of the findings

As for much qualitative research, there are limitations in understanding how to apply these findings to wider populations in Kilifi, across Kenya and elsewhere [40]. To strengthen this wider relevance, we designed the study to include individuals who were all relatively typical of local residents in this part of Kenya, with the notable exception of their greater exposure to and understanding of health research processes through earlier engagement activities with KWTRP. In this study, KCRs generally reflect demographic and socioeconomic features of wider population more accurately than students, since secondary school pupils in Kenya are a minority of the adolescents in this age group. For both groups, it seems likely that their relatively high levels of confidence in researchers (including some understanding of research review processes) influenced views on the acceptability of involving children in research and therefore also in assessing the role that children and adolescents should have in decision-making. At the same time, few studies have brought the voices of people directly involved in research activities in a LMIC setting to bear on these ethical questions, and the pre-existing relationships facilitated open conversations, arguably making it easier for participants to adopt critical stances.

### Why should children and adolescents be involved in decision-making?

The primary driver for promoting independent decision making by children and adolescents was discussed in relation to supporting or being in line with a ‘natural’ process of acquiring autonomy during growth and development, linked to views that children and adolescents should be listened to, and that involvement in decision-making about their own participation was an opportunity for a child or young person to learn more, as a pedagogical activity. Further, it was felt that children and adolescents should be involved in discussions about participation, where at all possible, because not to do so would violate their will – mentally and sometimes physically. In discussing emerging independence, parents in this consultation often talked about the need to ‘sit down and talk’ to children and adolescents to build understanding of any proposed research, why it was important and what would happen. This process was seen as one that would allow the child or young person to contribute to and accept a decision in ways that would avoid the harm of ‘violation’. Ideas about the wrongness of ‘violation’ were expressed most strongly when discussing the impossibility of forcing children and adolescents to participate in research against their will, unless important and otherwise unavailable health benefits were included. Of note, this right to refuse was recognised at a much younger age than the equivalent right to independently decide to participate, as is currently reflected in most ethics guidelines [3].

Participants’ views on why children and adolescents should be involved in decision-making are strongly in line with some of the principles argued for in the ethics literature, particularly that of respect for personhood for all children and adolescents, and the value of supporting development of their decision-making capacities. Views from this study that deciding to join a research project to contribute to a wider social value would be ‘good’ (as long as their own welfare is not negatively impacted) supported the development of the proposal in the Nuffield Council report that contributing to social goods are seen as an important opportunity for children and adolescents, not just for adults, and therefore could be considered part of a welfare argument in support of their participation. Emerging ideas about the pedagogical role of involving children in decision-making in the Nuffield Council working group were also supported by these findings [2].

### Should children’s and adolescents’ views on participation ever be overruled by their parents?

For participants in Kilifi, the fear that children and adolescents might make ‘bad’ decisions about research participation on their own countered parent’s desires to build their child’s independence over time as a normal part of growth. A ‘bad’ decision was primarily one likely to negatively impact welfare, again highlighting the importance of welfare considerations across this consultation. For this reason, it was argued that parents should exercise control over the decisions made. At the same time, there are caveats to participants’ arguments for overall parental control based on a welfare principle.

The first was made by many participants themselves, referencing a ‘generational shift’ linked to rapid social change. According to this argument, adolescents particularly may develop earlier competence for decision-making than previous generations. Wider access to education, including science, and social media may promote understanding of the issues at stake in making these decisions. This ‘fuzziness’ around where greatest competence for decision-making might lie led to strong recommendations around joint decision-making processes, drawing on both the parents’ and young person’s experience and knowledge. A second caveat that could be made, but was not raised in these discussions by KCRs and students, is that arguments about parents’ final control of decision-making is based on an assumption that they would always act in the interests of the individual child or young person, even if at times they may be mistaken in their assessment. In practice, such an assumption might not always hold true.

### When should children and adolescents be involved in decision-making?

Our findings echo those of Alderson (2017), looking at children’s competence in relation to decision-making about medical care, when she notes that ‘competence is not age or ability related, but depends on each child’s experience and confidence, on the child-parents relationships and values, and whether or not they are used to sharing knowledge, risk-taking and control over decisions’ [21] (p5). In this way, in Kilifi, recommendations around the extent of children and adolescents’ involvement in decision-making was associated with levels of independence from parents and perceptions of maturity, linked to but not dependent on age. Maturity was also seen as strongly influenced by aspects of the child or young person’s normal life, including exposure to formal schooling, levels of normal responsibility and independence in life, and there was no agreement on specific age categories that could be reliably associated with maturity. In any case, competence in making a decision was seen as a function of the complexity of the decision, particularly understanding of risks, benefits and social value of the research proposed. These interrelated influences of maturity linked to age, context and type of decision worked constantly across all discussions. Overall, increasing age and maturity (for example, being able to understand and think through implications of decisions in a stable manner), greater exposure to normal life responsibilities seen as equivalent to the research proposed, and more educational opportunities would increase a child or young person’s capacity to make decisions independently of (and potentially in opposition to) their parents. More complex research and greater risks or burdens of participation, with less individual health benefits, would limit their potential for independence.

The current practice of using age to assess competence for decision-making in many national and international ethics guidelines is a pragmatic solution to a complex situation of emerging competence. Our findings support Cheah and Parker’s (2014) proposal that using age is not only inaccurate but may often be overly restrictive where the context of children and adolescents’ lives suggests they will be able to make good decisions about research participation, particularly for minimal risk studies [12]. While we are making this argument from an LMIC setting, it is likely that this is the case in many other situations. The gender of children and adolescents may also play a role in decision-making dynamics in this and other traditionally patrilineal settings, needing careful attention to counter potential inequities for girls, young women and mothers.

In Kenya, the National Acquired Immune Deficiency Syndrome (AIDS) and Sexually Transmitted Infection (STI) Control Programme (NASCOP)/KEMRI guidelines [13] expands the definition of mature minors to include persons aged 15 years or older living apart from their parents or guardian and financially independent. The guidelines state that researchers should seek informed consent from emancipated or mature minors as opposed to assent, and that this takes away the requirement for parental/guardian consent. In addition, in Kenya and South Africa adolescents from 15 and 16 years of age, respectively, who are sexually active and seen as at risk for sexually transmitted infections, can make these decisions independently with Independent Research Ethics Board (IRB) approval [28]. In this way research ethics guidelines seem to both recognise the need to take account of social realities in relation to the age at which adolescents can make independent decisions, and continue to rely on age as a ‘cut off’ to indicate when both consent and assent processes should be used.

### How should children and adolescents be involved in decision-making?

Since age on its own is not a good reflection of maturity or competence, we have argued that a stronger approach is to view children or adolescents and their parents as making joint decisions about research participation. The Nuffield Council report in 2015 and the CIOMS guidelines in 2016 both highlight the value of a ‘co-consent’ process for older children and adolescents and their parents or guardians, rather than one of ‘assent and consent’ [49].

This recommendation, while important, also has a notable limitation. Joint decision-making processes may be influenced by power dynamics within families, so that children, adolescents and parents (particularly mothers in our setting) might still be unable to exercise control. The facilitation of family discussions would be key, requiring skills and time to recognise and manage potential challenges to fairness. In doing so, the requirement would be for researchers to support children and adolescents’ autonomy to the extent needed and to promote positive intra-family relations in the long term, unless there are clear reasons not to do this, towards the welfare of the child. Researchers may not have this skill set, and the resources needed to ensure fairness may be significantly greater than currently in place in many research contexts in LMICs. This challenge is critical since it would be important not to present important barriers to research involving children and adolescents in LMICs, given the urgent need for progress in reducing currently high burdens of disease and disability in this population. More research is critically important to understand what effective joint decision-making processes might look like in different settings, and what resources would be needed to support them.

### The importance of strengthening community-wide research literacy

We have described how unrealistic hopes and fear about research participation importantly influenced participants’ views on the acceptability of involving children in research and on the levels of independence that children and adolescents should have in decision-making about participation. Given this, where community members have relatively low familiarity with health research, as is common in LMIC settings (and others), wider community engagement strategies to build public understanding of research would be an important foundation to developing effective family decision-making processes. In our experience, effective engagement with communities about research needs attention to building relationships between research staff and local residents as well communication [50]. As the findings of this study also showed, the development of trust in these relationships is an important determinant of community understanding and the acceptability of research. At the same time, we noted the importance of ensuing that neither too much nor too little trust is placed on researchers in decision-making about children’s involvement in research, particularly for the potentially more complex processes involved in joint decision-making.

## Conclusion

This study in a largely rural coastal area of Kenya shows strong support from an LMIC setting for the idea that older children and adolescents should have a significant role in decision-making about their own participation in research. In most cases, KCRs and students involved in the consultation reasoned that adolescents should have a major or primary control over decision-making, where the balance of control relied on interrelated factors of the nature of the decision and individual competence to decide, influenced by considerations such as maturity, personality, normal decision making responsibilities and independence and exposure to science and research. The common emerging recommendation of a need to ‘sit down and talk’ seems closely aligned to an idea of decision-making through a parent-child dyad, supported by an external person to provide technical information and support.

While further research expanding diversity of context would be important to take forwards these findings, these findings show resonance with those reported from the UK and other high income settings on the acceptability of children and adolescents participating in at least some types of research; and the importance of actively involving these groups in making decisions about their own participation. There were strong concerns to protect and promote the short and long term welfare of individual children and adolescents within this process; and recognition that research on children’s health was important to supporting a wider population of children in future. Differences between the daily lives of children and adolescents in different contexts and intra-family dynamics are important considerations in assessing children’s capacity to make their own decisions about research participation. Research going forwards should address effective processes for context-specific joint consent processes for adolescents and their parents/guardians that takes account of the skills needed to facilitate a fair process and the burdens this might pose to overstretched research resources in LMICs.

## Additional files

Additional file 1: Focus group discussion guide for KEMRI Community Representatives. (DOCX 16 kb)

Additional file 2: Focus group discussion guide for students. (DOCX 17 kb)

## Data Availability

The datasets analysed during the current study are available from the KEMRI Wellcome Trust Research Programme Data Governance Committee through the corresponding author, on reasonable request.

## Abbreviations

AIDS: Acquired immune deficiency syndrome
CIOMS: Council for international organizations of medical science
HIV/AIDS: Human Immunodeficiency Virus/ Acquired Immune Deficiency Syndrome
IRB: Institutional review board
KCR: Kenya medical research institute community representative
KEMRI: Kenya medical research institute
KWTRP: Kenya Medical Research Institute Wellcome Trust Research Programme
LMIC: Low-to-middle income country
MacCAT-CR: MacArthur Competence assessment tool for clinical research
NASCOP: National Acquired Immune Deficiency Syndrome (AIDS) and Sexually Transmitted Infection (STI) Control Programme
RCPCH: Royal College of Paediatric and Child Health
SCC: Scientific Steering Committee, KEMRI
SEP: Schools Education Programme, KWTRP
STI: Sexually Transmitted Infection
UK: United Kingdom
UNCRC: United Nations Convention on the Rights of the Child
USA: United States of America
