# Supplementary material for: Who should decide about children’s and adolescents’ participation in health research? The views of children and adults in rural Kenya
Source: BMC Med Ethics. 2019 Jun 14;20:41. doi: 10.1186/s12910-019-0375-9 (PMC6570858; doi:10.1186/s12910-019-0375-9)
Supplement: Supplementary file 2 — Focus group discussion guide for students. (DOCX 17 kb) [file 12910_2019_375_MOESM2_ESM.docx]

**Additional file 2 : Focus group discussion guide for students**

**Background**

KEMRI Wellcome Trust Research Programme researchers are working in partnership with a UK-based organisation called The Nuffield Council on Bioethics to find out more about what children and young people think about taking part in health research. This is part of a project to look at ethical issues that come up when carrying out clinical research with children and young people – from babies up to 17 year olds - how can we do the research in the best possible way? The aim of the project is to produce a report to advise researchers on these ethical issues and ways of handling them. Although the report will be produced in the UK, the aim is to ensure that it is relevant to research in other countries in the world. For this reason, we are keen to listen to your views on how research that involves children and young people should be carried out.

In collaboration with the Kilifi County Education Officer, we have chosen four secondary schools in the County to visit as part of this project. They have been chosen on the basis of their involvement in the KEMRI Wellcome Trust Research Programme Schools Education Programme last year. In each school, we will meet with a group of students (about 6) for about one hour to listen to their views on research involving children and young people.

Although we will seek the advice of class teachers to identify possible participants for the groups, participation will be completely voluntary. Students who participate are also free not to discuss any subjects they don’t wish to talk about. Their views will be recorded anonymously, that is, by using codes rather than real names in any report so that anything participants say cannot be linked to them personally in future. As long as all the participants agree, we will voice record the group discussions to help us to capture all the views put forwards, but these will be permanently deleted once summaries of the discussions have been made.

In our work we are using the term ‘clinical research’ to mean research projects carried out in places like hospitals or clinics, where the main aim is to obtain new knowledge that will help improve the health care of all children in the future. This means that the research does not aim to improve the health of each participant in the study directly, although in some clinical research this may happen. Some clinical research studies will be closely related to the child’s treatment, whereas others will be quite separate and may involve healthy children or additional procedures. Examples are studies where researchers: only ask questions about a person’s health; only do blood tests (e.g. taking half a teaspoon); or try out new treatments to see how well they work - these generally also require taking small blood samples.

Many of the treatments and vaccines we use today were developed in this way through research done in the past. But children are very different from adults in the kinds of health problems they have and in the way their bodies handle different treatments. This means that research in children is important because otherwise it will not be possible to develop good new treatments for children in future. At the same time, clinical research in children raises some difficulties. While adults may choose to undergo any inconvenience, discomfort and potential risks that may be involved in clinical research, it is much harder for parents to make such decisions on behalf of their children. Many people feel that children should be protected from anything difficult as much as possible, but if children do not participate in research, how will we ensure they can be well treated if they become unwell in future?

In all the questions we will discuss in this group, there aren’t any ‘right’ or ‘wrong’ answers – we’re interested in knowing *what* you think and in finding out more about *why* you think it. Thank you for taking part!

**Feelings about taking part in research**

1. If you were asked, how would you/do you feel about participating in clinical research? What might you feel pleased, annoyed or worried about, and why? [Look for ‘hopes and concerns’]

- [Probe for direct experience] For anyone, is this on the basis of your own experience of participating in research in the past? If so, what was that research about, what did it involve and how old were you?
- [For any worries] What could be done to deal with any worries? (E.g. ways in which the research might be carried out that would feel better/more comfortable for children?)

**Influence of type of research**

1. Do you think your views would be different for different types of research? Earlier, we talked about research that involved only asking questions, or only taking blood, or also trying out new treatments (including some blood sampling) to see if they worked. Do the differences between these types of research change the way you think about participating? In what way? Are there other differences in the type of research that would change your point of view?

**Influence of age of child** (introduce and follow up in Q 5 & 6)

1. Would the age of the child asked to participate make a difference to you? If so, what kinds of ages would make a difference?

- *[Probe where needed*] For example, most of you in this group are between (aa) and (bb) years – what if the children needed in the research were much younger than you, or older than you? What difference would this make, why?
- What is it about those ages/age groups that make a difference?

**Who should make the decision about taking part?**

1. Who do you think should decide whether you take part in clinical research? (E.g. just you? You & your parents/guardians together? Your doctor& your parents? Someone else?) Why?

- What do you think should happen if you want to take part in research but your parents or guardians disagree with you? Why?
- What if you don’t want to take part in research, but your parents or guardians think you should? Why?
- What if your parents don’t agree with each other about this?
- Where studies are taking place only in schools, with the support of the DEO, does this make a difference to who should make the decision? (*Probe for role of teachers vs parents and of students; probe for studies that only include asking questions and those that include taking finger prick blood or urine/stool samples)*

1. Influences on making decisions:

- We talked earlier about how issues might be different for children of different ages – how would this affect your views on who should make the decision about taking part? (e.g. babies, young children or older children – see responses to Q3)
- What about the gender of the child? Would that make a difference & how? Any relationship to age e.g. boys considered more able to make independent decisions than girls of same age, or vice versa?
- Do you think religion plays a role in making a decision on child participation? If so, in what way?

**Should children participate in research that will not benefit them as individuals? [If time]**

1. If you were told that the research probably wouldn’t help you but might help other children in the future, would you still take part? Why/why not?

- [Linked to Q3] Would the age (of the child) make a difference? i.e. if the child asked was older or younger than you, would this make it seem more or less reasonable? In what way?
- Would the type of study make a difference?
